# COVID-19 Vaccine Acceptance Among Pregnant and Lactating Women in Saudi Arabia

**DOI:** 10.7759/cureus.32133

**Published:** 2022-12-02

**Authors:** Amal S Bagalb, Dlal Almazrou, Amani A Albraiki, Latifa I Alflaih, Lama O Bamunif

**Affiliations:** 1 Pharmaceutical Care Services, King Saud Medical City, Riyadh, SAU; 2 Quality, Saudi Central Board for Accreditation of Healthcare Institutions, Riyadh, SAU; 3 Department of Pharmacy Practice, College of Pharmacy, Princess Nourah Bint Abdulrahman University, Riyadh, SAU

**Keywords:** vaccine acceptance, pregnant and lactating, pregnant females, covid-19 vaccine, vaccine safety

## Abstract

Background

The acceptance of vaccines among pregnant and breastfeeding women is vital to alleviate the risk of contracting and transmitting coronavirus disease 2019 (COVID-19). Therefore, we aimed to assess the COVID-19 vaccine acceptance among pregnant and breastfeeding/lactating women and the factors associated with the acceptance of the COVID-19 vaccine in Saudi Arabia.

Methodology

A cross-sectional survey was conducted among pregnant and breastfeeding women living in Saudi Arabia. A 23-item, self-administered questionnaire was used to assess the COVID-19 vaccine acceptance among pregnant or lactating women.

Results

A total of 160 (53.3%) pregnant women and 140 (46.7%) breastfeeding/lactating mothers participated in the study. When the participants were asked, “Have you been vaccinated or are you planning to take the vaccine during pregnancy or breastfeeding/lactation?” 164 (54.7%) responded with “Yes.” When compared with breastfeeding/lactating mothers (54, 38.6%), pregnant women had more concerns about the efficacy and safety of COVID-19 vaccination (77 (48.1%); p = 0.043). The probability of rejecting COVID-19 vaccination was higher among breastfeeding/lactating women with a lower education level than those with a tertiary education level (odds ratio = 3.42, confidence interval = 1.24, 9.45, p = 0.018).

Conclusions

This study reported high acceptance of COVID-19 vaccination in a sample of breastfeeding/lactating women. Concern about vaccine safety among many pregnant women was the major reason for hesitancy. Factors such as low education, concerns regarding the efficacy and safety of the vaccine, and doctors’ (e.g., obstetrician and gynecologist) recommendations for the COVID-19 vaccine were associated with vaccine acceptance.

## Introduction

Coronavirus disease 2019 (COVID-19) was identified in December 2019 in Wuhan City, China [1]. Since then, it has become an unforeseen challenge for the global health system. Many approaches to tackle community transmission have halted almost every social and economic activity in numerous countries [2-4]. Vaccination is recognized as the most effective approach to protecting against infectious diseases. As of October 2021, a total of 194 COVID-19 candidate vaccines are under development, with 126 currently being tested in clinical trials [5]. Eight vaccines have already been approved for full use [6]. COVID-19 vaccines vary in their efficacies, for instance, Comirnaty (m-RNA-based vaccine) and Moderna (m-RNA-1273) have the highest efficacies of 95% and 94.5%, respectively [7-10]. Similarly, the reported efficacies of Sputnik V, Convidecia, and CoronaVac are 91.6%, 65.28%, and 50.38%, respectively.

People are always more inclined toward safe and effective vaccines [11]. In addition, perceptions about the safety of vaccines and sociodemographic characteristics also affect acceptance rates. Previous reports inform that the vaccine acceptance rate is relatively higher in males than in females [12]. A recent report from 19 countries demonstrated that the potential acceptance rate of a COVID-19 vaccine ranges from 54.8% (Russia) to 88.6% (China) [11]. However, this report lacks data about the acceptance rate of the COVID-19 vaccine and its determinants in the Middle East. Since the beginning of the pandemic and as of October 15, 2021, a total of 547,807 cases and 8,755 deaths have been reported in the Kingdom of Saudi Arabia (KSA) [13]. Healthcare professionals in the KSA have administered 44.3 million COVID-19 vaccine doses [13].

The risk of severe disease and emergency presentations is relatively more in pregnant women than in non-pregnant women [14-16]. Therefore, pregnant women are classified as a high-risk cohort for COVID-19 infection [17]. Clinical trials on COVID-19 vaccines generated very limited evidence on efficacy and safety for pregnant and breastfeeding/lactating women. Studying the acceptance of the COVID-19 vaccine is an urgent requirement as the World Health Organization (WHO) listed vaccine hesitancy as one of the top 10 threats to global health, even preceding the COVID-19 pandemic [18,19]. The acceptance of the vaccine among pregnant and breastfeeding women in the KSA is vital to alleviate the risk of contracting and transmitting COVID-19, thus reducing the risk of hospitalizations due to COVID-19 [20]. It is crucial to ascertain the factors influencing the acceptance rate for the COVID-19 vaccine among pregnant and breastfeeding women in the KSA. Previous reports about the COVID-19 vaccine acceptance in the KSA did not focus on pregnant and breastfeeding/lactating women [21,22]. Therefore, we aimed to assess the COVID-19 vaccine acceptance among pregnant and breastfeeding/lactating women and the factors associated with the acceptance of the COVID-19 vaccine in the KSA.

## Materials and methods

A cross-sectional survey using an anonymous questionnaire was conducted between November 2021 and February 2022. We included pregnant or lactating women aged 18 years and over and excluded women who were not pregnant and were not breastfeeding. The study was approved by King Saud Medical City, Ministry of Health (approval number: H1RI-30-Nov21-01).

A non-probability snowball sampling technique was used to recruit the study participants from the maternity department of the tertiary care setting. A 23-item, self-administered questionnaire after face and content validation was distributed to the pregnant or lactating women currently living in the KSA. We prepared the initial draft of the questionnaire after adopting questions from previous reports [23-25], expert opinions from senior healthcare professionals, and feedback obtained from females who visited the maternity department. The face validity, utility, and feasibility of the draft questionnaire were ascertained via a pilot sample of 10 females (e.g., pregnant and lactating women) who visited the maternity department. The questionnaire was prepared to be finished within 20 minutes. The participants of a pilot sample evaluated individual items and highlighted those that were deemed inappropriate in regard to phrasing and applicability. We also reviewed the questionnaire after collating feedback from the pilot sample to ensure that all questions were easy to comprehend and complete. The questionnaire comprised questions related to demographics (age, education, and occupation) and general questions related to the acceptability of the COVID-19 vaccine, including perceived benefits and barriers to vaccination. Following the completion of the study questionnaire, participants were asked to share the invitations with their friends and colleagues. Further, all participants were asked to share the questionnaire with their email contacts and social networks, including WhatsApp, Twitter, and Facebook. The study objectives were included on the first page of the questionnaire under the consent statement to provide general information to the study participants.

To elicit a response from 50% of respondents, with a 95% confidence level and a 5% margin of error, a sample size of 377 was estimated. The sample size was calculated by an online sample size calculator [26]. Descriptive statistics were used to determine the frequency and percentages of categorical variables. The determinants or factors of the COVID-19 vaccine were assessed in a two-stage process. First, a binary logistic regression was applied to examine the associations between each individual explanatory variable such as sociodemographic and health-related characteristics, as well as response acceptance (willingness to receive a COVID-19 vaccine). We performed univariate and multivariate logistic regression tests. All variables presenting a p-value <0.25 were included in an adjusted analysis in the second stage of the analysis. Each independent variable was attributed an odds ratio (OR) with 95% confidence intervals (95% CI) and significance (p). A p-value of less than 0.05 was considered statistically significant. Data analysis was carried out using SPSS version 24 (IBM Corp., Armonk, NY, USA).

## Results

Out of the 377 questionnaires distributed among the target population, 300 were completed and returned: 160 (53.3%) pregnant women and 140 (46.7%) breastfeeding/lactating mothers. The response rate was 80%. The majority of the respondents (115, 38.3%) were between the age of 25-29 years, mostly pregnant women (73, 45.6%). While breastfeeding/lactating mothers were predominantly 30-34 years old (n = 53, 38%). Moreover, the highest proportion of the participants had a university education (238, 79.3%) and did not have chronic diseases such as hypertension and diabetes mellitus (Table 1).

**Table 1:** Study characteristics of overall data and between pregnant and lactating women. All categorical data are presented as n (%). P < 0.05 was considered statistically significant.

Variables	Overall, n (%) = 300	Pregnant, n (%) = 160 (53.3)	Breastfeeding/Lactating, n (%) = 140 (46.7)	P-value
Age, years				<0.001
18–24	12 (4)	10 (6.3)	2 (1.4)	
25–29	115 (38.3)	73 (45.6)	42 (30)	
30–34	104 (34.7)	51 (31.9)	53 (37.9)	
35–40	51 (17)	25 (15.6)	26 (18.6)	
>40	18 (6)	1 (0.6)	17 (12.1)	
Highest education				0.023
Intermediate or lower school	17 (5.7)	5 (3.1)	12 (8.6)	
Secondary school	45 (15)	19 (11.9)	26 (18.6)	
University	238 (79.3)	136 (85)	102 (72.8)	
Occupation				0.594
Student	6 (2)	3 (1.9)	3 (2.1)	
Housewife	137 (45.7)	68 (42.4)	69 (49.3)	
General employee (healthcare-related job)	82 (27.3)	48 (30)	34 (24.3)	
General employee (non-healthcare-related job)	67 (22.3)	38 (23.8)	29 (20.7)	
Online employee	8 (2.7)	3 (1.9)	5 (3.6)	
Any chronic disease (e.g., diabetes, high blood pressure, or others)				0.013
Yes	24 (8)	7 (4.4)	17 (12.1)	
No	276 (92)	153 (95.6)	123 (87.9)	

When the participants in this study were asked, “Have you been vaccinated or are you planning to take the vaccine during pregnancy or breastfeeding/lactation?” 164 (54.7%) responded with “Yes.” Among these respondents, the proportion of breastfeeding/lactating women who said yes to the question was statistically higher (88, 62.9%) than that of the pregnant women group (76, 47.5%) (p = 0.0046).

The participants were also asked questions related to the potential benefits and safety of receiving COVID-19 vaccines. A high proportion (141, 47%) of the overall participants remained neutral when they were asked the question about the potential of COVID-19 to cause harm. A higher proportion of this number were pregnant women (87, 54.4%), and 54 (38.6%) were breastfeeding/lactating mothers, with a p-value of 0.007. When compared with breastfeeding/lactating mothers (54, 38.6%), pregnant women had more concerns about the efficacy and safety of COVID-19 vaccination (77, 48.1%) (p = 0.043). Moreover, over half of the total number of participants (154, 58.1%) did not feel safe due to the absence of data about COVID-19 vaccine safety. A higher proportion of this cohort were pregnant women (93, 58.1%) than breastfeeding/lactating mothers (61, 43.6%), with a p-value of 0.017.

A total number of 127 (42.4%) respondents agreed that the possible side effects of COVID-19 could interfere with their usual activities, and most of these participants were pregnant women (76 (47.5%), p = 0.014). However, 60 (42.9%) of those who are breastfeeding/lactating disagreed. With regard to a preference for using alternative remedies, the majority of pregnant women (59, 36.9%) preferred traditional medicines compared to orthodox vaccines. In contrast, 56 (40%) breastfeeding/lactating mothers believed in synthetic vaccines. The participant’s responses to the survey questions are presented in Table 2.

**Table 2:** COVID-19 vaccine acceptance rates and comparisons of the answers between pregnant and lactating women. COVID-19 = coronavirus disease 2019

Variables	Overall, n (%)	Pregnant, n (%)	Breastfeeding/Lactating, n (%)	P-value
Q1: Have you been vaccinated or are you planning to take the vaccine during pregnancy or breastfeeding/lactation?				0.046
Yes	164 (54.7)	76 (47.5)	88 (62.9)	
No	41 (13.7)	25 (15.6)	16 (11.4)	
Not Sure	19 (6.3)	10 (6.3)	9 (6.4)	
I was vaccinated before pregnancy or starting breastfeeding/lactation	76 (25.3)	49 (30.6)	27 (19.3)	
Q2: Vaccination helps reduce the risk of virus infection				0.825
Agree	211 (70.3)	114 (71.2)	97 (69.3)	
Neutral	50 (16.7)	27 (16.9)	23 (16.4)	
Disagree	39 (13)	19 (11.9)	20 (14.3)	
Q3: Vaccination will ease complications of the disease				0.614
Agree	207 (69)	113 (70.6)	94 (67.2)	
Neutral	64 (21.3)	34 (21.3)	30 (21.4)	
Disagree	29 (9.7)	13 (8.1)	16 (11.4)	
Q4: The vaccine will help to provide long-term immunity				0.854
Agree	121 (40.3)	64 (40)	57 (40.7)	
Neutral	128 (42.7)	67 (41.9)	61 (43.6)	
Disagree	51 (17)	29 (18.1)	22 (15.7)	
Q5: COVİD-19 vaccine carries the possibility of harm for me				0.007
Agree	70 (23.3)	37 (23.1)	33 (23.6)	
Neutral	141 (47)	87 (54.4)	54 (38.6)	
Disagree	89 (29.7)	36 (22.5)	53 (37.9)	
Q6: I am concerned about the efficacy and safety of COVID-19 vaccination in pregnant and breastfeeding/lactating women				0.043
Agree	131 (43.7)	77 (48.1)	54 (38.6)	
Neutral	104 (34.7)	57 (35.6)	47 (33.6)	
Disagree	65 (21.6)	26 (16.3)	39 (27.9)	
Q7: I am afraid of injections				0.895
Agree	51 (17)	27 (16.9)	24 (17.1)	
Neutral	46 (15.3)	26 (16.3)	20 (14.3)	
Disagree	203 (67.7)	107 (66.9)	96 (68.6)	
Q8: I believe that COVID-19 is not a serious disease				0.127
Agree	56 (18.7)	32 (20)	24 (17.1)	
Neutral	104 (34.7)	62 (38.8)	42 (30)	
Disagree	140 (46.7)	66 (41.3)	74 (52.9)	
Q9: I don’t need vaccination because I have a low risk for COVID-19 infection				0.124
Agree	40 (13.3)	26 (16.3)	14 (10)	
Neutral	80 (26.7)	46 (28.7)	34 (24.3)	
Disagree	180 (60)	88 (55)	92 (65.7)	
Q10: I am worried that the possible side effects of COVID-19 vaccination would interfere with my usual activities				0.014
Agree	127 (42.4)	76 (47.5)	51 (36.4)	
Neutral	70 (23.3)	41 (25.6)	29 (20.7)	
Disagree	103 (34.3)	43 (26.9)	60 (42.9)	
Q11: I believe in natural or traditional remedies more than synthetic vaccines				0.071
Agree	103 (34.3)	59 (36.9)	44 (31.4)	
Neutral	97 (32.4)	57 (35.6)	40 (28.6)	
Disagree	100 (33.3)	44 (27.5)	56 (40)	
Q12: I don’t need vaccination because I’m young and healthy				0.164
Agree	51 (17)	30 (18.4)	21 (15)	
Neutral	80 (26.7)	48 (30)	32 (22.9)	
Disagree	169 (56.3)	82 (51.2)	87 (62.1)	
Q13: I am afraid or do not feel safe due to the lack of data about COVID-19 vaccine safety in pregnant and lactating women				0.017
Agree	154 (51.3)	93 (58.1)	61 (43.6)	
Neutral	61 (20.3)	32 (20)	29 (20.7)	
Disagree	85 (28.3)	35 (21.9)	50 (35.7)	
Q14: I am against all types of vaccines				0.589
Agree	36 (12)	22 (13.8)	14 (10)	
Neutral	52 (17.3)	28 (17.5)	24 (17.1)	
Disagree	212 (70.7)	110 (68.8)	102 (72.9)	
Q15: I would accept the COVID-19 vaccine if adequate information about its safety and effectiveness is confirmed				0.742
Q16: I would only take the COVID-19 vaccine if the vaccine is taken by many of the population (especially pregnant and breastfeeding/lactating women)				0.175
Agree	199 (66.3)	102 (63.7)	97 (69.3)	
Neutral	42 (14)	28 (17.5)	14 (10)	
Disagree	59 (19.7)	30 (18.8)	29 (20.7)	
Q17: I would agree to be vaccinated against COVID-19 only if it is recommended by my obstetrics and gynecology doctor				0.418
Agree	209 (69.7)	114 (71.3)	95 (67.9)	
Neutral	37 (12.3)	16 (10)	21 (15)	
Disagree	54 (18)	30 (18.8)	24 (17.1)	
Q18: I would only take the COVID-19 vaccine if mandatory vaccination is required by the government				0.305
Agree	196 (65.3)	104 (65)	92 (65.7)	
Neutral	43 (14.3)	27 (16.9)	16 (11.4)	
Disagree	61 (20.3)	29 (18.1)	32 (22.9)	

The results of the univariate analysis are illustrated in Table 3. Out of the 18 variables tested in the univariate analysis, only five were statistically significant in the multivariate logistic regression. The results of the final model demonstrate that two factors were significantly associated with accepting COVID-19 vaccines by pregnant women. Further, three factors were found to be associated with COVID-19 vaccination acceptance among pregnant women in KSA.

**Table 3:** Univariate analysis for predictors of COVID-19 vaccine acceptance among pregnant and lactating women. COVID-19 = coronavirus disease 2019

Variables	Pregnant (n = 160)			Breastfeeding/Lactating (n = 140)		
	Odds ratio	95% confidence interval	P-value	Odds ratio	95% confidence interval	P-value
Age (reference >40 years)						
18–24	8.0	1.01-63.96	0.050	0.2	0.04-0.68	0.014
25–29	62.0	8.60-447.13	<0.001	2.4	1.25-4.56	0.009
30–34	37.0	5.08-269.69	<0.001	3.8	2.09-7.08	<0.001
35–40	17.0	2.26-127.74	0.006	1.5	0.72-2.96	0.292
Highest education (reference: University)						
Intermediate or Lower school	0.03	0.01-0.09	<0.001	0.08	0.04-0.17	<0.001
Secondary school	0.15	0.09-0.26	<0.001	0.20	0.12-0.33	<0.001
Occupation (reference: Online employee)						
Student	3.0	0.31-28.84	0.341	1.0	0.20-4.95	1.000
Housewife	51.0	7.05-369.04	<0.001	17.0	5.31-54.47	<0.001
General employee (healthcare-related job)	38.0	5.22-276.77	<0.001	11.0	3.37-35.87	<0.001
General employee (non-healthcare related job)	32.0	4.37-234.18	0.001	8.3	2.52-27.60	0.001
Any chronic disease (ref: No)	0.04	0.02-0.10	<0.001	0.14	0.08-0.24	<0.001
Vaccination helps reduce the risk of virus infection (reference: disagree)						
Agree	11.2	5.68-22.19	<0.001	7.2	3.92-13.11	<0.001
Neutral	1.7	0.73-3.81	0.226	1.4	0.68-2.97	0.356
Vaccination will ease complications of the disease (reference: disagree)						
Agree	19.2	7.81-47.18	<0.001	7.5	3.97-13.99	<0.001
Neutral	4.8	1.83-12.58	0.001	2.0	0.97-4.12	0.061
The vaccine will help to provide long-term immunity (reference: disagree)						
Agree	3.23	1.88-5.57	<0.001	3.20	1.79-5.71	<0.001
Neutral	3.19	1.81-5.38	<0.001	3.47	1.95-6.16	<0.001
COVİD-19 vaccine carries the possibility of harm for me (reference: disagree)						
Agree	0.65	0.38-1.11	0.112	0.52	0.32-0.87	0.012
Neutral	2.03	1.35-3.06	0.001	1.09	0.73-1.64	0.677
I am concerned about the efficacy and safety of COVID-19 vaccination in pregnant and breastfeeding/lactating women (reference: disagree)						
Agree	2.32	1.41-3.82	0.001	1.09	0.69-1.72	0.726
Neutral	2.36	1.44-3.89	0.001	1.20	0.77-1.88	0.426
I am afraid of injections (reference: disagree)						
Agree	0.25	0.16-0.40	<0.001	0.18	0.11-0.32	<0.001
Neutral	0.24	0.15-0.39	<0.001	0.22	0.13-0.37	<0.001
I believe that COVID-19 is not a serious disease (reference: disagree)						
Agree	0.36	0.22-0.60	<0.001	0.27	0.16-0.45	<0.001
Neutral	0.79	0.54-1.17	0.240	0.53	0.35-0.81	0.003
I don’t need vaccination because I have a low risk for COVID-19 infection (reference: disagree)						
Agree	0.25	0.15-0.41	<0.001	0.11	0.06-0.22	<0.001
Neutral	0.40	0.26-0.60	<0.001	0.33	0.21-0.51	<0.001
I am worried that the possible side effects of COVID-19 vaccination would interfere with my usual activities (reference: disagree)						
Agree	1.46	0.96-2.22	0.076	0.73	0.48-1.11	0.142
Neutral	0.92	0.58-1.46	0.722	0.48	0.30-0.78	0.003
I believe in natural or traditional remedies more than synthetic vaccines (reference: disagree)						
Agree	1.38	0.87-2.17	0.170	0.75	0.49-1.16	0.192
Neutral	1.53	0.98-2.40	0.061	0.65	0.41-1.01	0.058
I don’t need vaccination because I’m young and healthy (reference: disagree)						
Agree	0.26	0.16-0.43	<0.001	0.18	0.10-0.38	<0.001
Neutral	0.45	0.30-0.68	<0.001	0.30	0.19-0.47	<0.001
I am afraid or do not feel safe due to the lack of data about COVID-19 vaccine safety in pregnant and lactating women (reference: disagree)						
Agree	1.97	1.29-3.01	0.002	1.00	0.66-1.51	1.000
Neutral	0.94	0.57-1.54	0.800	0.56	0.34-0.91	0.018
I am against all types of vaccines (reference: disagree)						
Agree	0.19	0.11-0.32	<0.001	0.13	0.07-0.23	<0.001
Neutral	0.27	0.17-0.42	<0.001	0.18	0.11-0.31	<0.001
I would accept the COVID-19 vaccine if adequate information about its safety and effectiveness are confirmed (reference: disagree)						
Agree	14.86	6.91-31.94	<0.001	14.14	6.57-30.44	<0.001
Neutral	2.00	0.81-4.96	0.134	1.29	0.48-3.45	0.618
I would only take the COVID-19 vaccine if the vaccine is taken by many of the population (especially pregnant and breastfeeding/lactating women) (reference: disagree)						
Agree	5.47	3.26-9.17	<0.001	4.47	2.72-7.36	<0.001
Neutral	0.88	0.44-1.77	0.724	0.58	0.28-1.22	0.149
I would agree to be vaccinated against COVID-19 only if it is recommended by my obstetrics and gynecology doctor (reference: disagree)						
Agree	6.19	3.65-10.49	<0.001	7.08	3.87-12.96	<0.001
Neutral	0.63	0.28-1.38	0.244	1.50	0.72-3.11	0.277
I would only take the COVID-19 vaccine if mandatory vaccination is required by the government (reference: disagree)						
Agree	4.05	2.51-6.53	<0.001	2.96	1.90-4.62	<0.001
Neutral	0.91	0.49-1.68	0.752	0.46	0.23-0.92	0.027

Pregnant women who agreed with the statement “I do not need vaccination because I am young and healthy” were approximately three times more likely not to accept COVID-19 vaccination than those who disagreed (OR = 2.54, CI = 1.11, 5.75, p = 0.027). Further, those who believed that “I would only take the COVID-19 vaccine if the vaccine is taken by many of the population (especially pregnant women)” are 61% less likely to accept the COVID-19 vaccination compared to pregnant women who did not agree with the statement (OR = 0.39, CI = 0.19, 0.81, p = 0012).

The probability of rejecting COVID-19 vaccination was higher among breastfeeding/lactating women with a lower education level than those with a tertiary education level (OR = 3.42, CI = 1.24, 9.45, p = 0.018). Further, breastfeeding/lactating women who were concerned about the efficacy and safety of COVID-19 vaccination are nearly three times more likely not to accept COVID-19 than those who were not (OR = 2.92, CI = 1.09, 7.79, p = 0.032. Breastfeeding/lactating women who agreed to be vaccinated against COVID-19 only if it is recommended by an obstetrics and gynecology doctor were 64% less likely to reject COVID-19 vaccination compared with those who disagreed (OR = 0.34, CI = 0.15, 0.77, p = 0.010). The results of the final model are illustrated in Table 4.

**Table 4:** Multivariate logistic regression for predictors of COVID-19 vaccine acceptance among pregnant and lactating women. Reference is “Not required vaccination” for all categories. COVID-19 = coronavirus disease 2019

Variables	Pregnant (n = 160)			Breastfeeding/Lactating (n = 140)		
	Odds ratio	95% confidence interval	P-value	Odds ratio	95% confidence interval	P-value
I do not need vaccination because I’m young and healthy	2.53	1.11-5.75	0.027	1.95	0.71-5.39	0.199
I would only take the COVID-19 vaccine if the vaccine is taken by many of the population (especially pregnant and breastfeeding/lactating women)	0.39	0.19-0.81	0.012	0.85	0.37-1.93	0.697
Highest education	1.35	0.42-4.36	0.621	3.42	1.24-9.45	0.018
I am concerned about the efficacy and safety of COVID-19 vaccination in pregnant and breastfeeding/lactating women	0.99	0.39-2.49	0.982	2.92	1.09-7.79	0.032
I would agree to be vaccinated against COVID-19 only if it is recommended by my obstetrics and gynecology doctor	1.02	0.46-2.29	0.953	0.34	0.15-0.77	0.010

## Discussion

This study reports the first data on the rate and factors associated with accepting COVID-19 vaccination among pregnant and breastfeeding/lactating women in the KSA. We found that the COVID-19 vaccination was accepted by over half of the total participants. Specifically, we observed pregnant women were more willing to receive the vaccination compared with breastfeeding/lactating mothers. Factors associated with accepting the COVID-19 vaccination among pregnant women were having a belief that someone does not need it because she is young and healthy, and the feeling that a person would only receive the vaccine if it was taken by many pregnant women. Similarly, three factors were found to be associated with the willingness to receive COVID-19 by breastfeeding/lactating women. The first was education level, followed by concerns about COVID-19 safety and efficacy, and the third factor was the willingness to receive the vaccine based on the recommendation of a specialist as it could be because of breastfeeding/lactating women’s trust in their doctors. Our findings can guide interventions for promoting COVID-19 vaccination among this population.

The acceptance rate of COVID-19 vaccination among pregnant and breastfeeding women is widely reported in the literature [27-29]. What we obtained in our study was similar to what was found in a systematic review and meta-analysis of studies from four continents [30]. According to the study, 49% of pregnant women indicated a willingness to receive the COVID-19 vaccine [30]. In another study by Ayhan and his colleagues [24], 37% of pregnant women indicated their willingness to receive the vaccine if it was recommended by a specialist. In the KSA and many other countries, there is an absence of research on the effects of the COVID-19 vaccine in pregnancy, and as demonstrated in our study, there is a higher proportion of indecision because of possible harm. This limitation could partly affect the acceptance rate among the pregnant women population. Therefore, there is a need for data on the safety of COVID-19 on pregnant and breastfeeding women that could guide the development of strategies for improving COVID-19 uptake in the KSA.

Identifying factors that could be barriers to receiving COVID-19 vaccinations can guide the development and implementation of interventions for enhancing the vaccination rate in the community. In this study, we found that pregnant women who agree that they do not need vaccination because they are young and healthy are three times more likely to reject COVID-19 vaccination. This finding could be due to the lack of adequate public knowledge about the vaccine in the KSA. This poor knowledge is evident in a recent study conducted among the general population in KSA, which shows that 30-40% of people lacked information about COVID-19 vaccination among the young population and pregnant women [31]. Thus, measures to improve COVID-19 vaccination reuptake should target this knowledge deficit.

We found that having a lower education level is a predictor of rejecting COVID-19 vaccination among breastfeeding/lactating mothers in the KSA. Lower education level has been widely reported as one of the barriers to receiving COVID-19 vaccination among the public in the KSA [32,33]. Promotional campaigns on improving awareness and reuptake of COVID-19 vaccinations should target breastfeeding/lactating women with a lower education level in the community. Efficacy/safety concern about COVID-19 vaccination was another factor associated with willingness to receive the vaccination among breastfeeding/pregnant women. A possible explanation could be the fear of potential harm to their young babies through COVID-19 vaccination [27]. According to a global survey, 32.7% of breastfeeding women report that they would expect to see additional information/evidence about COVID-19 vaccination in children [29]. Therefore, addressing this fear through educational programs among breastfeeding mothers could increase the vaccination rate, and consequently, stop the pandemic in the KSA.

This study identified gaps that can prevent the actualization of the WHO COVID-19 immunization target of 70% [34]. Targeting the identified factors during the implementation of the educational awareness campaign can improve knowledge and subsequent COVID-19 acceptance rate in the KSA. Our findings suggest the need for improved patient education about COVID-19 vaccination among pregnant and breastfeeding/lactating women during medical consultations at healthcare facilities and pharmacies in the KSA.

This is the first study of its kind that assessed factors associated with the acceptance of the COVID-19 vaccine among Saudi pregnant and breastfeeding/lactating women. Nonetheless, this study also has certain limitations. One of the important study limitations is that the sample of 377 females (e.g., pregnant and lactating women) was not possible due to limited visits of this population, particularly during the COVID-19 period at our maternity department. Further, this was a cross-sectional survey performed at a single tertiary care setting, and, therefore, cannot inform about causality, and the findings may not be generalized to the entire group of pregnant and lactating women in Saudi Arabia. Therefore, there is a need for conducting a national cross-sectional study with a large sample size via probability sampling among Saudi pregnant and breastfeeding/lactating women regarding their perception and attitude toward COVID-19 vaccine acceptance. In addition, future research can investigate the impact of the provision of education regarding the efficacy and safety of the COVID-19 vaccine on the acceptance of vaccines among pregnant and breastfeeding/lactating women.

## Conclusions

This study reported high acceptance of COVID-19 vaccination in a sample of breastfeeding/lactating women. Concerns about vaccine safety among many pregnant women were the major reason for hesitancy. Factors such as low education, concerns regarding the efficacy and safety of the vaccine, and doctors’ (e.g., obstetrician and gynecologist) recommendations for COVID-vaccine were associated with the COVID-19 vaccine acceptance. Due to the low acceptance rate among pregnant women, it is crucial to address concerns among hesitant cohorts by building trust in vaccine safety and effectiveness through the provision of evidence-based information about the COVID-19 vaccine.

## Appendices

**Table 5:** Study questionnaire. COVID-19 = coronavirus disease 2019

Age (in years)	18–24
	25–29
	30–34
	35–40
	>40
Higher education	Intermediate or Lower school
	Secondary school
	University
Occupation	Student
	Housewife
	General employee (healthcare-related job)
	General employee (non-healthcare-related job)
	Online employee
Do you have any chronic disease (e.g., diabetes, high blood pressure, or others)	Yes, No
Have you been vaccinated or are you planning to take the vaccine during pregnancy or breastfeeding/lactation?	Yes, No, Not sure, I was vaccinated before pregnancy or starting breastfeeding/lactation
Vaccination will ease complications of the disease	Agree, Neutral, Disagree
The vaccine will help to provide long-term immunity	Agree, Neutral, Disagree
The COVID-19 vaccine carries the possibility of harm for me	Agree, Neutral, Disagree
Vaccination helps reduce the risk of virus infection	Agree, Neutral, Disagree
I am concerned about the efficacy and safety of COVID-19 vaccination in pregnant and breastfeeding/lactating women	Agree, Neutral, Disagree
I am afraid of injections	Agree, Neutral, Disagree
I believe that COVID-19 is not a serious disease	Agree, Neutral, Disagree
I don’t need vaccination because I have a low risk for COVID-19 infection	Agree, Neutral, Disagree
I am worried that the possible side effects of COVID-19 vaccination would interfere with my usual activities	Agree, Neutral, Disagree
I believe in natural or traditional remedies more than synthetic vaccines	Agree, Neutral, Disagree
I don’t need vaccination because I’m young and healthy	Agree, Neutral, Disagree
I am afraid or do not feel safe due to the lack of data about COVID-19 vaccine safety in pregnant and lactating women	Agree, Neutral, Disagree
I am against all types of vaccines	Agree, Neutral, Disagree
I would accept the COVID-19 vaccine if adequate information about its safety and effectiveness are confirmed	Agree, Neutral, Disagree
I would only take the COVID-19 vaccine if the vaccine is taken by many of the population (especially pregnant and breastfeeding/lactating women)	Agree, Neutral, Disagree
I would agree to be vaccinated against COVID-19 only if it is recommended by my obstetrics and gynecology doctor	Agree, Neutral, Disagree
I would only take the COVID-19 vaccine if mandatory vaccination is required by the government	Agree, Neutral, Disagree
